# Household Hints and Recipes

**Published:** 1878-01

**Authors:** 


### Household Hints & Recipes.

LIQUID SHOE-POLISH.—The following is from a German source: Dissolve 3½ oz. of shellac in a half-pint of alcohol. Rub smooth 25 grains of lamp-black with 6 drachms of cod-liver-oil, and mix. A few drops are to be applied to the leather with a sponge.

---

TO MAKE SHOES WATER-TIGHT.—The following receipt is from the *Droguisten Zeitung*: A litre of boiled linseed-oil, 125 gm. of mutton suet, 46 gm. of wax, and 32 gm. resin, are melted together on a charcoal fire, under constant stirring, and the melted mixture applied to the well cleaned and dried shoes. The leather retains its full elasticity, and becomes absolutely impervious to water.

---

TO CLEAN VESSELS THAT HAVE CONTAINED KEROSENE.—Wash the vessel with thin milk of lime, which will form an emulsion with the petroleum and remove all traces of it. By washing a second time with milk of lime, and a very small quantity of chloride of lime, and allowing the liquid to remain in the vessel about an hour, and then washing it with cold water, the smell may be removed. If the milk of lime be used warm instead of cold, the operation will be rendered much shorter.

---

A GOOD VARNISH.—Varnish made with alcohol will get dull and spongy by the evaporation of the alcohol, which leaves water in the varnish, as all commercial alcohol contains water. Take thin sheet gelatin, cut it into strips, and put it in the varnish; it will absorb most of the water, and the varnish can be used clear and bright till the last drop. The gelatin will get quite soft; it can then be taken and dried and used again. "I have used this plan," says the writer, "for the last two years in photographic varnish, and have never had to throw away one drop."

---

TO REMOVE SILVER STAINS FROM FABRICS.—This process is said to be especially successful in removing spots from materials which have been several times washed. First prepare a saturated solution of cloride of copper, dip the spotted piece in the solution, and allow it to remain some minutes, or according to the character of the stains. Then rub the stains with crystal hyposulphite of soda. When neutral cloride of copper is used, the color of the stuff does not change. This process can be repeated.—*The Photographic News.*

